# Soft Tissue Chondroma of the Oral Cavity: An Extremely Rare Tumour Localized on the Hard Palate

**DOI:** 10.1155/2014/414861

**Published:** 2014-03-04

**Authors:** Paolo Vescovi, Marco Meleti, Elisabetta Merigo, Maddalena Manfredi, Domenico Corradi, Ilaria Giovannacci, Tito Poli, Samir Nammour

**Affiliations:** ^1^Center of Oral Laser Surgery and Oral Pathology, Dental School, Department of Biomedical, Biotechnological and Translational Sciences, University of Parma, Via Gramsci 14, 43125 Parma, Italy; ^2^Section of Pathology and Laboratory Medicine, Department of Biomedical, Biotechnological and Translational Sciences, University of Parma, Italy; ^3^Section of Maxillofacial Surgery, Department of Biomedical, Biotechnological and Translational Sciences, University of Parma, Italy; ^4^University of Liège, Belgium

## Abstract

Chondromas are benign cartilaginous tumors usually localized within the tubular bones of the extremities. Soft tissue chondromas (STCs) are rare and only few cases have been reported in the oral cavity. The present case documents the exceptional finding of a 12-year-standing STC of the hard palate of a 63-year-old man. The tumor measured approximately 6 cm in its larger size and it was radically excised through the use of a quantic resonance molecular (QRM) lancet. No recurrence was observed during 1-year follow-up. A concise review of the relevant literature is included in the present paper.

## 1. Introduction

Soft tissues chondromas (STCs) are rare, benign tumors occurring in extraosseous and extrasynovial locations. By definition, these tumours are not attached to bone, intra-articular synovium, or periosteum [1].

STCs are usually diagnosed within the soft tissues of the hands and feet, the fingers being frequently affected [1, 2]. Other localizations include the dura, larynx, skin, and fallopian tube [1, 3–6]. STCs rarely occur in the oral cavity [7]. Subsites where these tumours have more frequently been detected include the tongue, the cheek mucosa, both hard and soft palate, the edentulous ridge, and the masticatory space [7–14].

Oral STCs are apparently more common in the third and fourth decades [7–14].

From the clinical point of view, these neoplasms usually present as nonsymptomatic, slow-growing, and well-defined nodules expanding into the surrounding tissues [1, 15].

STCs are predominantly composed of adult type hyaline cartilage, devoid of other differentiated elements, except osseous, fibrous, and/or myxoid stroma [1].

With specific regard to oral STCs, the tongue is the most common site of occurrence. Sera et al. described an elastic, semicircular nodule with a diameter of approximately 5 mm localized on the dorsum of the tongue. The authors also reported the presence of other 45 cases in the literature of fibrochondroma of the tongue [8].

Onodera et al. reported one of the 5 cases in the literature of STCs of the cheek and de Riu et al. described a case of a STC of the masticatory space [9, 14]. This lesion was diagnosed as a result of an incidental report of a radiopaque mass localized in the temporomandibular joint seen on a standard X-ray of the cervical spine [14].

To the best of our knowledge, only five cases of STC of the palate have been reported in the literature, one of these being associated with cleft palate [16–20].

No reliable theories have been put forward to clarify the etiology and pathogenesis of oral STCs even though an origin from aberrant embryonal cartilage or metaplastic changes due to chronic inflammation or trauma may be hypothesized [8, 21].

A genetic relation to the pathogenesis of this condition, in particular to chromosome 12 and chromosomes 6 and 11, has been hypothesized in two studies [22, 23].

The present case documents the exceptional finding of a STC of the hard palate altogether with a concise review of the literature.

## 2. Case Report

A 63-year-old man was referred to the Center of Oral Laser Surgery and Oral Pathology, Dental School, University of Parma, Italy, with a diagnosis at admission of a “fibroma of the hard palate.” Patient complained of a slow-growing 12-year-standing lesion that was starting to interfere with chewing and speech. He stated that he never underwent oral examination before because of a superstitious and popular attitude, considering the lesion as a punishment from God.

Familiar anamnesis was negative for chondromas and social history did not highlight relevant aspects.

**Figure 1:**
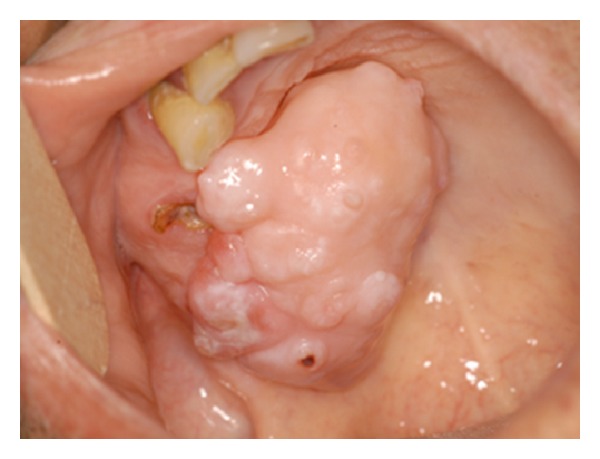
Clinical aspect of the soft tissue chondroma. The lesion appears as a nodular mass, approximately measuring 6 cm in its larger size, localized on the palatal side of the edentulous ridge.

Clinical evaluation revealed the presence of a nodular mass, approximately measuring 6 cm in its larger size, localized on the palatal side of an edentulous ridge corresponding to the 1.3–1.7 region (Figure 1). The lesion was pedunculated, uniformly colored with an irregular surface. A remnant root, most probably of the 1.5, was present on the close proximity of the peduncle. On palpation, a hard-fibrous consistence as well as presence of focal hard tissue could be appreciated. Pain and ulceration were absent. No history of systemic diseases, drugs administration, local trauma, irradiation, or chronic inflammation was recorded on anamnesis and the patient was otherwise healthy.

Radiological examination did not show presence of bone involvement or intraneoplastic calcifications.

**Figure 2:**
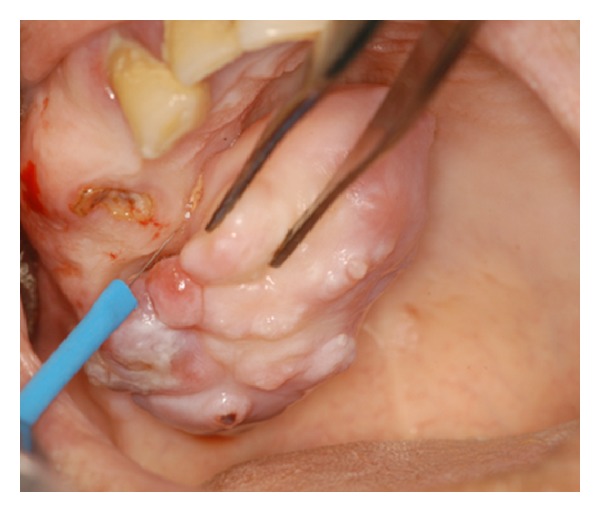
Removal of the lesion through the use of quantic molecular resonance (QRM) scalpel.

**Figure 3:**
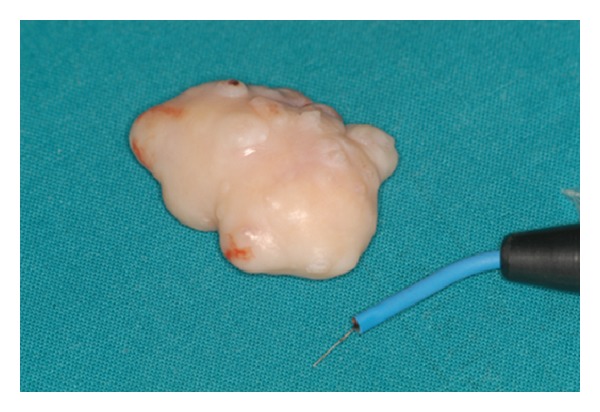
Aspect of the surgical specimen (Bladion©, length of cutting tool = 4 cm).

Working diagnoses included minor salivary gland neoplasm and peripheral ossifying/cementifying fibroma. Because of the pedunculated nature of the lesion, an excisional biopsy, using a molecular quantic resonance scalpel (Bladion©, length of cutting tool = 4 cm), was performed (Figures 2 and 3). Hemostasis was obtained through the use of the Bladion© coagulative mode. The root was extracted during the surgical procedure. Postsurgical macroscopic examination confirmed the absence of underlying bone involvement. Postsurgical course was normal and no recurrence was observed during one-year follow-up.

### 2.1. Histopathological Evaluation

After the excision, the specimen was immediately fixed in a 10% buffered formalin, cut into several tissue slices, and embedded in paraffin tissue blocks, according to conventional methods. Five-*μ*m-thick sections were obtained for hematoxylin and eosin, periodic acid-Schiff, and Alcian Blue. Additional histological sections were cut for immunohistochemical analysis which was performed according to the manufacturers' protocols. Negative control procedures were performed by omitting the primary antibody, in each case.

**Figure 4:**
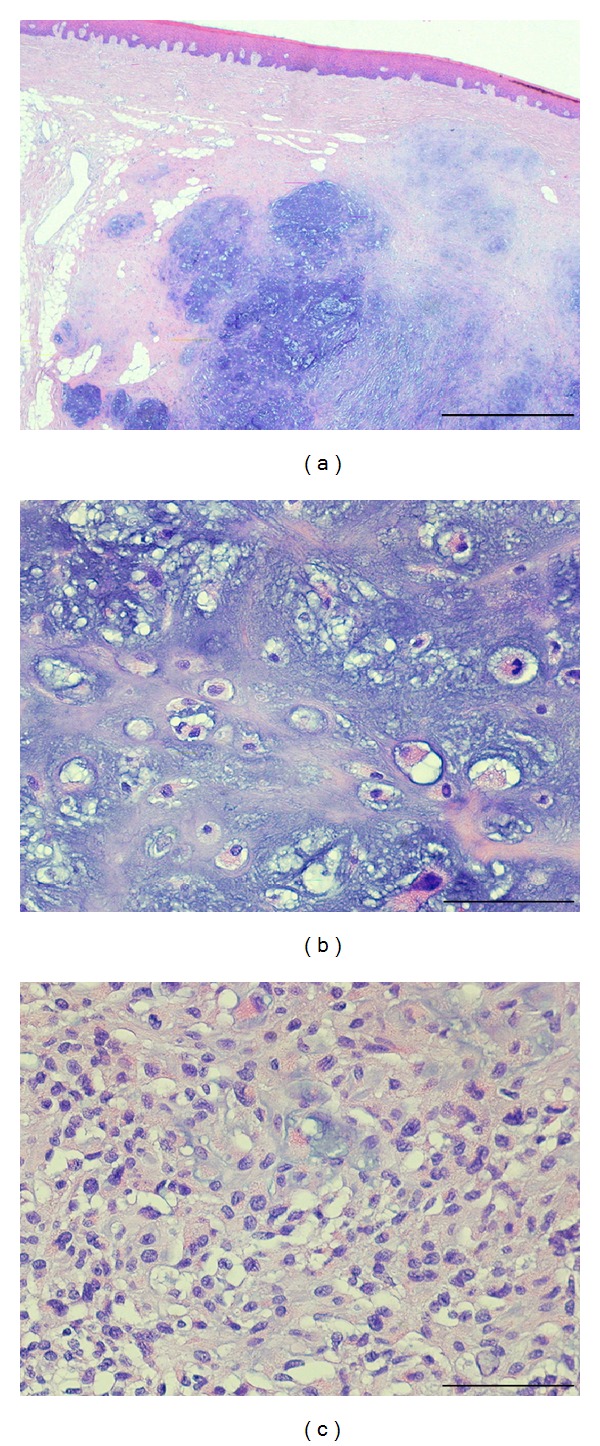
(a) Low-magnification view of the soft tissue chondroma located in the subepitelial hard palate. It shows a multilobular pattern with large blue areas, constituted of cartilaginous tissue, surrounded by a densely sclerotic fibrous tissue. No signs of ulceration are present. Haematoxilin-eosin, original magnification ×2, bar is 1 mm. (b) The cartilaginous cells are immersed in an extremely abundant matrix and sometimes are binucleated (arrow). Haematoxilin-eosin, original magnification ×20, bar is 100 *μ*m. (c) Focally, this lesion is constituted of smaller elements characterised by either oval or reniform nucleus and a slightly eosinophilic cytoplasm. Haematoxilin-eosin, original magnification ×20, bar is 100 *μ*m.

On the cut section, this lesion appeared multinodular and blue-greyish with slightly well-defined edges. This appearance corresponded, histologically, to a cartilaginous tumour characterised by multiple nodules composed of mature chondrocytes placed in an abundant blue chondroid matrix (Figure 4(a)). These cells, focally, showed a mild degree of pleomorphism with some binucleations (Figure 4(b)). In addition, sometimes these aggregates were made of smaller round elements with either reniform or oval nuclei (Figure 4(c)). The background of this multinodular pattern was intensely sclerotic with some fibroblasts. There were no signs of either infiltration or ulceration of the overlying epithelium. Immunohistochemically, the lesional cells were diffusely positive for vimentin and S100 and always negative for cytokeratin pool, epithelial membrane antigen (EMA), desmin, smooth muscle actin, caldesmon, p63, human melanoma black-45 (HMB-45), and calponin.

## 3. Discussion

Swellings on the hard palate are very common and may depend on a variety of factors including minor salivary glands neoplasms, traumatic, or idiopathic fibrous reactions as well as acute and chronic infectious processes.

Very few cases of STCs limited to the hard palate were reported [16, 17]. Pathogenesis and clinical behavior of oral STCs are still poorly understood and the knowledge of these neoplasms is mainly based on the evaluation of few documented cases [7–14, 16]. A possible genetic influence has recently been outlined [22, 23]. In contrast to the case reported here the case of STC of the hard palate described by Nehete et al. was associated with complete cleft palate and it was present from birth. The patient presented with an asymptomatic globular swelling of approximately 3 cm that completely filled the cleft. The congenital nature of the tumour led the authors to hypothesize an origin from embryologic remnants. The tumour was excised and cleft palate repair was performed by pushback palatoplasty. On the contrary, the case reported here developed during the adult life of the patient and this aspect can hardly fit with an embryologic disturbance. Similarly to the cases reported in the literature, also in our case, surgical excision seems to be effective to treat the tumour. No recurrence was noticed after 2 years of follow-up [16].

The majority of patients with a diagnosis of STCs are middle-aged with a range between 26 and 60 years [1, 9]. A slightly higher incidence between female patients is outlined even though others report a male preponderance [1, 7–14, 16]. The length of time between the appearance of first symptoms and treatment varied from 1 to 31 years, with a median of 8.4 years for STCs of the cheek mucosa [9].

According to Onodera et al. intraoral STCs mainly involve the lateral borders and the dorsum of the tongue, even though cases have been described on the soft palate, cheek, tonsil, flabby ridge beneath dentures, and the masticatory space [9, 16].

For tumors of the tongue, a possible pathogenetic mechanism involving the paraphysiologic cartilaginous tissue of the lingual septum (so-called “knorpelinsel”) might be considered [21]. Such a hypothesis may also explain the high incidence of the recently described ectomesenchymal chondromyxoid tumors at this site [24].

Radiological appearance of oral STCs is rather unspecific. These neoplasms may appear as a well-demarcated, lobulated mass with peripheral or central calcifications often curvilinear in nature [1]. However, not one of the previous radiological features was observed in the present case.

Diagnosis of oral STCs may be difficult and usually requires histopathological confirmation. Several tumors of soft tissue may display cartilaginous differentiation as primary phenomenon (e.g., ectomesenchymal chondromyxoid tumor; extra skeletal myxoid and mesenchymal chondrosarcoma) or as a secondary metaplastic process (e.g., malignant nerve sheath tumors, oral malignant melanoma) [1, 24, 25]. Benign lipomatous (chondrolipoma) and fibromatous tumors (calcifying aponeurotic fibroma) as well myositis ossificans may also show metaplastic cartilage [26–28].

According to Nayler and Heim, STCs are microscopically composed of lobules of mature, adult hyaline cartilage, with chondrocytic cells often growing in clusters [1]. One-third of cases may display extensive calcification, particularly in the centre of tumour lobules [1]. Presence of abundant myxoid matrix with immature cells may sometimes be observed. STCs cells positively stain for S100 protein [1, 7–14, 16].

Ten to 15% percent of tumors may recur locally after surgical excision [1]. In the present case, no recurrence has been observed after one-year follow-up.

Differently from osseous and synovial cartilaginous tumors, transformation to chondrosarcoma has not been described in extraskeletal chondromas [1, 8].

## References

[1] Nayler S, Heim S, Fletcher DM, Unni KK, Mertens F (2002). Soft tissue condroma. Tumors of soft tissue and bone. *WHO Classification of Tumors (Chondro-Osseous Tumours)*.

[2] Dahlin DC, Salvador AH (1974). Cartilaginous tumors of the soft tissues of the hands and feet. *Mayo Clinic Proceedings*.

[3] Brownlee RD, Sevick RJ, Rewcastle NB, Tranmer BI (1997). Radiologic-pathologic correlation. Intracranial chondroma. *The American Journal of Neuroradiology*.

[4] Devaney KO, Ferlito A, Silver CE (1995). Cartilaginous tumors of the larynx. *Annals of Otology, Rhinology and Laryngology*.

[5] Ando K, Goto Y, Hirabayashi N, Matsumoto Y, Ohashi M (1995). Cutaneous cartilaginous tumor. *Dermatologic Surgery*.

[6] Han JY, Han HS, Kim YB, Kim JM, Chu YC (2002). Extraskeletal chondroma of the fallopian tube. *Journal of Korean Medical Science*.

[7] Munro JM, Singh MP (1990). Chondroma of the tongue. Report of a case and a consideration of the histogenesis of such lesions. *Archives of Pathology and Laboratory Medicine*.

[8] Sera H, Shimoda T, Ozeki S, Honda T (2005). A case of chondroma of the tongue. *International Journal of Oral and Maxillofacial Surgery*.

[9] Onodera K, Xu H, Kimizuka S, Echigo S, Ooya K (2005). Chondroma of the cheek: a case report. *International Journal of Oral and Maxillofacial Surgery*.

[10] Blum MR, Danford M, Speight PM (1993). Soft tissue chondroma of the cheek. *Journal of Oral Pathology and Medicine*.

[11] Gardner DG, Paterson JC (1968). Chondroma or metaplastic chondrosis of soft palate. *Oral Surgery, Oral Medicine, Oral Pathology*.

[12] Weller CV (1923). The incidence and histopathology of bone and cartilage in the tonsil. *Annals of Otology, Rhinology, and Laryngology*.

[13] Cutright DE (1972). Osseous and chondromatous metaplasia caused by dentures. *Oral Surgery, Oral Medicine, Oral Pathology*.

[14] de Riu G, Meloni SM, Gobbi R, Contini M, Tullio A (2007). Soft-tissue chondroma of the masticatory space. *International Journal of Oral and Maxillofacial Surgery*.

[15] Chung EB, Enzinger FM (1978). Chondroma of soft parts. *Cancer*.

[16] Nehete R, Nehete A, Sankalecha S (2012). Soft tissue chondroma of hard palate associated with cleft palate. *Indian Journal of Plastic Surgery*.

[17] Kawanoa T, Yanamotoa S, Kawasakia G, Mizunoa A, Fujita S, Ikedab T (2011). Soft tissue chondroma of the hard palate: a case report. *Asian Journal of Oral and Maxillofacial Surgery*.

[18] Ramanathan K, Keat TC, Singh H (1970). Chondroma of the palate. Case report. *Australian Dental Journal*.

[19] Snyder SR, Merkow LP (1973). Benign chondroma of the palate: report of case. *Journal of Oral Surgery*.

[20] Ide F (2006). Chondromyxoid tumor of palate. *Journal of Oral Pathology and Medicine*.

[21] Roy JJ, Klein HZ, Tipton DL (1970). Osteochondroma of the tongue. *Archives of Pathology*.

[22] Cin PD, Qi H, Sciot R, van den Berghe H (1997). Involvement of chromosomes 6 and 11 in a soft tissue chondroma. *Cancer Genetics and Cytogenetics*.

[23] Shadan FF, Mascarello JT, Newbury RO, Dennis T, Spallone P, Stock AD (2000). Supernumerary ring chromosomes derived from the long arm of chromosome 12 as the primary cytogenetic anomaly in a rare soft tissue chondroma. *Cancer Genetics and Cytogenetics*.

[24] de Visscher JGAM, Kibbelaar RE, van der Waal I (2003). Ectomesenchymal chondromyxoid tumor of the anterior tongue. Report of two cases. *Oral Oncology*.

[25] Rosemberg AE, Heim S, Fletcher DM, Unni KK, Mertens F (2002). Extraskeletal osteosarcoma. Tumors of soft tissue and bone. *WHO Classification of Tumors (Chondro-Osseous Tumours)*.

[26] Maes A, Eulderink F (1989). Chondrolipoma of the tongue. *Histopathology*.

[27] Fetsch JF, Miettinen M (1998). Calcifying aponeurotic fibroma: a clinicopathologic study of 22 cases arising in uncommon sites. *Human Pathology*.

[28] Sarac S, Sennaroglu L, Hosal AS, Sozeri B (1999). Myositis ossificans in the neck. *European Archives of Oto-Rhino-Laryngology*.

